# Trigeminal neuropathic pain is alleviated by inhibition of Ca_v_3.3 T-type calcium channels in mice

**DOI:** 10.1080/19336950.2020.1859248

**Published:** 2020-12-23

**Authors:** Marena Montera, Aleyah Goins, Leos Cmarko, Norbert Weiss, Karin N. Westlund, Sascha R. A. Alles

**Affiliations:** a Department of Anesthesiology and Critical Care Medicine, University of New Mexico School of Medicine, Albuquerque, NM, USA; b Institute of Biology and Medical Genetics, First Faculty of Medicine, Charles University, Prague, Czech Republic; c Institute of Organic Chemistry and Biochemistry, Czech Academy of Sciences, Prague, Czech Republic

**Keywords:** Neuropathic pain, calcium channels, Cav3.3, trigeminal nerve injury, therapeutics

## Abstract

In this brief report, we demonstrate that the Ca_v_3.3 T-type voltage-gated calcium channel subtype is involved in our FRICT-ION model of chronic trigeminal neuropathic pain. We first showed that the *Cacna1i* gene encoding Ca_v_3.3 is significantly upregulated in whole trigeminal ganglia of FRICT-ION mice compared to controls at week 10 post-injury. We confirmed protein upregulation of Ca_v_3.3 compared to controls using Western blot analysis of whole trigeminal ganglia tissues. Finally, we demonstrated that intraperitoneal injection of a selective TAT-based Ca_v_3.3 blocking peptide in FRICT-ION mice significantly reduces Ca_v_3.3 protein expression at the peak anti-allodynic effect (4 hrs post-injection) of the attenuated neuropathic pain behavior. We also suggest that blockade of Ca_v_3.3 may be more effective in attenuating trigeminal neuropathic pain in female than male FRICT-ION mice. Therefore, blocking or attenuating Ca_v_3.3 function may be an effective strategy for the treatment of trigeminal neuropathic pain.

## Introduction

Chronic pain is experienced by one in five people globally. Opioids, which are very effective for acute pain, are not very effective for chronic pain. Therefore, the problem of chronic pain is compounded by opioid abuse. It is clear that more effective treatments are urgently needed.

One type of chronic pain that is particularly traumatic for patients is trigeminal neuropathic pain which is caused by inflammation and demyelination of the trigeminal nerve pathways. We have developed a highly robust, long-lasting, and easily induced animal model of trigeminal neuropathic pain that accurately depicts the kind of pain suffered by patients. We call this model FRICT-ION (Foramen Rotundum Inflammatory Constriction Trigeminal InfraOrbital Nerve) [1]. The FRICT-ION model persists through long time frames suitable for testing therapeutics for chronic pain (>6 months) [2]. An equivalent clinical trial model used in clinical drug testing is third molar extraction. This common procedure provides ready proof-of-concept, dose-ranging, and efficacy testing for pain therapeutics that are later effectively guided through FDA approvals [2,3].

In this study, we have analyzed the differential gene expression pattern of trigeminal ganglia (TG) from FRICT-ION mice compared to naïve mice and established a potential role for the Ca_v_3.3 T-type calcium channel. We performed behavioral pharmacology experiments using a selective Ca_v_3.3-blocking peptide and found that mechanical allodynia is alleviated in both male and female FRICT-ION mice. Furthermore, we also show that the Ca_v_3.3-blocking peptide appears to be more effective in female than male mice in alleviating mechanical allodynia in our model.

This study is the first to demonstrate a potential sex difference in the role of Ca_v_3.3 in trigeminal neuropathic pain. This may pave the way for the development of a novel therapeutic for the treatment of trigeminal neuropathic pain, especially in women.

## Materials and methods

### FRICT-ION model of trigeminal neuropathic pain

The procedures in this study were approved by the Institutional Animal Care and Use Committees of the University of New Mexico. All animals were housed in a well-ventilated rodent housing room (maintained at 20–22°C) with a reversed 10/14 h dark/light cycle so that testing could be performed in their active period. All animals were housed for 1 week before the experiments. All animals had access to food and water *ad libitum* throughout the duration of the experiment. Low soybean content normal chow diet was provided. All surgeries were completed in a sterile environment under a surgical microscope in mice anesthetized with isoflurane (2–5%). The FRICT-ION model was induced as previously described [1] in under 10 min per mouse in male and female BALBc (20 to 25 g; 8–10 weeks; Harlan Laboratories, Indianapolis, IN). Mechanical allodynia developed within 1 week post-surgery and persisted through >10 weeks as evaluated using von Frey filaments.

### Behavioral assays

Mechanical threshold of the whisker pad area was tested before and after surgery with a modified up/down method using a graded series of von Frey fiber filaments as described previously [1,4,5].

### RNA extraction and RNAseq

Male and female mice (8–10 weeks old) were subjected to the FRICT-ION model of trigeminal neuropathic pain. At 10 weeks post-injury, mice were euthanized by anesthetic overdose with pentobarbital (50 mg/kg) and both ipsilateral and contralateral whole trigeminal ganglia (TG) were removed. TG were washed immediately in PBS and stored at −80°C to preserve RNA. RNA was isolated using RNeasy Mini Kits (Qiagen, USA). Yield and quality of RNA were determined using a Nanodrop device. RNA was only used for further analysis if 260/280 ratio was ~2.0. Whole TG RNA samples from three FRICT-ION injured and three naïve mice were sent to Quick Biology (Pasadena, USA) for RNAseq library preparation, sequencing using the Illumina HiSeq 4000 at 40 million reads per sample and gene expression analysis. The reads were first mapped to the latest UCSC transcript set using Bowtie2 version 2.1.0 and the gene expression level was estimated using RSEM v1.2.15. Differentially expressed genes were identified using the edgeR program. Genes showing altered expression with p < 0.05 were considered differentially expressed.

### Peptide synthesis and administration

TAT-based cell-penetrating peptides used in this study were previously described (Cmarko and Weiss, Mol Brain 2020) and were synthesized by GenScript®. A Ca_v_3.3-blocking peptide called TAT-C3P (amino acid sequence GRKKRRQRRRPQEESNKEAREDAELDAEIELEMAQG, molecular weight 4311 g/mol) was administered to male and female mice via intraperitoneal injection at a dose of 10 mg/kg and monitored every hour for up to 5 h post-injection. For control experiments, a peptide called TAT-C3D (amino acid sequence GRKKRRQRRRPQAVSSPARSGEPLHALSPRGTARSP, molecular weight 4006 g/mol) was used which does not block Ca_v_3.3 channels. For all experiments, the peptides were dissolved in distilled water.

### Western blot

Twelve male and female mice (8–10 weeks old, six mice per sex) were subject to the FRICT-ION model of trigeminal neuropathic pain. In week 3 post-nerve injury, three mice of each sex were administered TAT-C3P peptide and the remaining mice were administered TAT-C3D following the methods described above. Four hours post-injection, mice were euthanized with pentobarbital (50 mg/kg) and both ipsilateral and contralateral TG were removed. TG were washed immediately in PBS and stored at −80°C.

The TG for each of the four groups were pooled together (n = 3) for protein extraction and homogenized using a pestle and 500 µL of 1x RIPA buffer (ThermoFisher Scientific, Cat# 89,900). Samples were put on a rocking shaker for 2 h and then centrifuged, and the supernatant removed to a new tube. Sample was assayed for total protein. (Bradford, ThermoFisher Scientific). Samples were then prepared for electrophoresis by mixing with 2x sample buffer and boiling for 5 min at 100°C for denaturation.

Proteins were loaded on a 12% Tris-Glycine polyacrylamide gradient gel (Bio-Rad) and transferred to a PVDF membrane (Millipore). Membrane was blocked for an hour with 5% nonfat milk in TBST buffer at room temperature and incubated at 4°C with anti-Ca_v_3.3 antibody overnight (Alomone Labs, Cat# ACC-009, diluted 1:200 in 2.5% milk in TBST). The membrane was subsequently washed with TBST and then incubated with anti-rabbit secondary with HRP for 1 h at room temperature (Abcam ab6721, diluted 1:1000 in TBST). The washing was repeated and then the blot developed with chemiluminescent substrate (ThermoFisher Scientific, Cat#32,106). The blot was then imaged using a Li-Cor Odyssey FC imaging system. Signal intensity was normalized to actin, which was used as a loading control (Abcam ab8227, 1:2000). Signal intensity was analyzed using ImageJ for comparisons.

### Data analysis

Males and females were analyzed separately. Whisker pad mechanical thresholds were averaged for FRICT-ION-injured mice receiving TAT-C3P or TAT-C3D peptides. The behavioral data were expressed as mean ± SEM using two-way analysis of variance (ANOVA) with post-hoc testing with Tukey’s multiple comparisons over time. A p-value of <0.05 was considered significant. Normalized fold change between TAT-C3P-peptide-treated and control TAT-C3D-peptide-treated mice was compared with t-tests for the Western blots. Statistical analysis of the transcript cluster-level data comparisons was done via paired t-tests.

## Results

### Cacna1i *is upregulated in trigeminal ganglia of FRICT-ION mice*

Previous studies have indicated that changes at the transcriptional level of the dorsal root or trigeminal ganglia may underlie the pathophysiology of neuropathic pain in several animal models [6–8]. Therefore, in order to determine the changes that may be occurring at the transcriptional level in FRICT-ION mice compared to control, we analyzed trigeminal ganglia (TG) gene expression by RNAseq 10 weeks after nerve injury. We found that the expression of the *Cacna1i* gene, encoding the Ca_v_3.3 ion channel, was increased by 40% (p < 0.05, paired t-test; Table 1). *Cacna1i* was one of only a few ion channel genes upregulated while most were downregulated at 10 weeks (Table 1). Genes encoding other channels upregulated 10 weeks after nerve injury included *Kcnh6, Clic4, Kcnj10, Hvcn1*, and *Kcnj16*.

**Table 1.** RNAseq analysis showing up- and down-regulated ion channel gene expression in TG isolated from FRICT-ION mice. The expression of all Cav3 subtypes, *Cacna1g, Cacna1h, Cacna1i* is indicated. *Cacna1i* expression increase is statistically significantly (p < 0.05, paired t-test), whereas that of *Cacna1g* and Cacna1h are not altered (p > 0.05, paired t-test) compared to naïve controls. Expression of housekeeping gene *Gapdh* is not significantly changed compared to controls (p > 0.05, paired t-test). TG were isolated at 10 weeks post-injury. This analysis is based on RNA obtained from whole TG tissue of three FRICT-ION and three naïve male mice. Percentage change with corresponding p-values is indicated to three significant figures

Gene symbol	Name	% change	p-value
Upregulated ion channel genes			
*Cacna1g*	calcium channel; voltage-dependent; T type; alpha 1 G subunit	35.6%	0.358
*Cacna1h*	calcium channel; voltage-dependent; T type; alpha 1 H subunit	10.9%	0.160
*Cacna1i*	calcium channel; voltage-dependent; alpha 1I subunit	39.8%	0.0276
*Gapdh*	glyceraldehyde-3-phosphate dehydrogenase	3.47%	0.590
*Kcnh6*	potassium voltage-gated channel; subfamily H (eag-related); member 6	20.3%	0.0449
*Clic4*	chloride intracellular channel 4 (mitochondrial)	25.1%	0.0208
*Kcnj10*	potassium inwardly-rectifying channel; subfamily J; member 10	37.0%	0.000219
*Hvcn1*	hydrogen voltage-gated channel 1	60.4%	0.0370
*Kcnj16*	potassium inwardly-rectifying channel; subfamily J; member 16	81.5%	0.0102
*Downregulated ion channel genes*			
*Kcnb2*	potassium voltage gated channel; Shab-related subfamily; member 2	−44.2%	0.0270
*Kcnv1*	potassium channel; subfamily V; member 1	−40.0%	0.0112
*Kcnh5*	potassium voltage-gated channel; subfamily H (eag-related); member 5	−35.3%	0.0194
*Kcnh7*	potassium voltage-gated channel; subfamily H (eag-related); member 7	−34.4%	0.0302
*Kcnmb2*	potassium large conductance calcium-activated channel; subfamily M; beta member 2	−32.0%	0.0468
*Scn1a*	sodium channel; voltage-gated; type I; alpha	−25.2%	0.00392
*Kcnq3*	potassium voltage-gated channel; subfamily Q; member 3	−21.2%	0.0321
*Kcna2*	potassium voltage-gated channel; shaker-related subfamily; member 2	−18.3%	0.0341
*Scn9a*	sodium channel; voltage-gated; type IX; alpha	−16.1%	0.0414
*Nalcn*	sodium leak channel; nonselective	−14.4%	0.0172
*Trpm7*	transient receptor potential cation channel; subfamily M; member 7	−13.6%	0.0171

### TAT-C3P, a Ca_v_3.3-selective blocking peptide, alleviates mechanical allodynia in FRICT-ION mice

**Figure 1.**
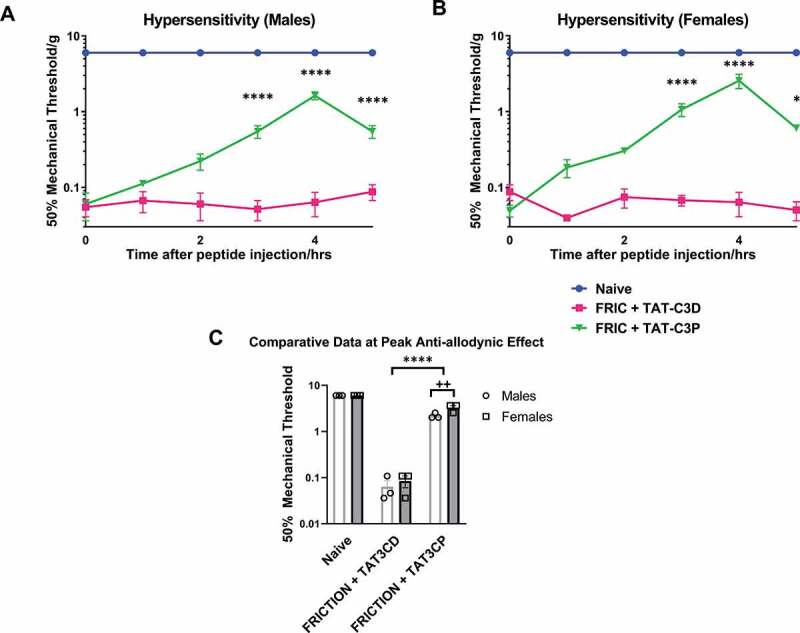
Effect of Ca_v_3.3-blocking peptide on mechanical allodynia in FRICT-ION mice. A Ca_v_3.3-blocking peptide, TAT-C3P, was administered to (A) male and (B) female mice via intraperitoneal injection at a dose of 10 mg/kg and monitored every hour for up to 5 h post-injection. For control experiments TAT-C3D was used, which does not block Ca_v_3.3 channels. (C) Effectiveness of 10 mg/kg Ca_v_3.3-blocking peptide at peak anti-allodynic effect (4 hrs post-injection) is compared versus naïve, TAT-C3D control, and versus male and female mice * p < 0.05 and ** p < 0.01 compared to FRIC+TAT-C3D, **** p < 0.0001 compared to FRIC+TAT-C3D, ++ p < 0.01 compared to males with FRIC+TAT-C3D. Lack of error in naïve behavioral measurements is because of a ceiling effect for mechanical threshold in these mice

Since Ca_v_3.3 was upregulated in the TG of FRICT-ION mice, we hypothesized that blocking this channel would alleviate neuropathic pain in our model. To test this we used the Ca_v_3.3-selective blocking peptide TAT-C3P, which is highly selective for Cav3.3 over other Cav3 subtypes [9]. We demonstrated a significant increase (p < 0.0001, 2-way ANOVA with *posthoc* Tukey’s test) in mechanical withdrawal thresholds in both male (Figure 1a) and female (Figure 1b) FRICT-ION mice. The control TAT-C3D peptide, which does not block Ca_v_3.3, produced no significant change (p > 0.05, 2-way ANOVA) in withdrawal thresholds in either male or female FRICT-ION mice (Figure 1a-b). We also unexpectedly found that blocking Ca_v_3.3 resulted in a more significant increase in withdrawal threshold in female than male mice. Comparing the peak anti-allodynic effect observed at the 4 h post-TAT-C3P injection time point, we show that female FRICT-ION mice withdrawal thresholds were significantly higher than male FRICT-ION mice withdrawal thresholds (Figure 1c; p < 0.01, 2-way ANOVA with *posthoc* Tukey’s test).

### Ca_v_3.3 protein levels are increased in TG of FRICT-ION mice compared to controls and these are lowered by TAT-C3P

**Figure 2.**
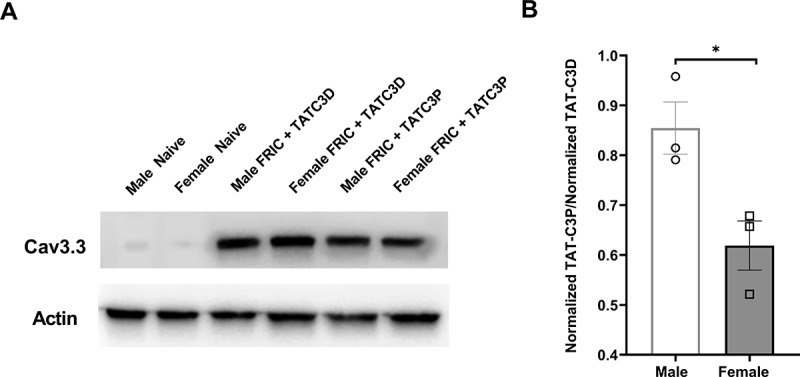
Protein levels of Ca_v_3.3 in male and female FRICT-ION mice following Ca_v_3.3-blocking peptide injection. (A) Ca_v_3.3 vs actin levels in whole TG lysates of male and female naïve, male and female FRICT-ION injected with control TAT-C3D peptide, and male and female FRICT-ION mice injected with Ca_v_3.3-blocking TAT-C3P peptide (n = 3 mice per group). (B) Normalized ratio of intensity of TAT-C3P-injected to control TAT-C3D-injected male and female FRICT-ION mice at the peak of the anti-allodynic effect (4 hrs post-injection). (*p < 0.05, t-test)

In order to determine whether Ca_v_3.3 protein levels were increased in TG of FRICT-ION mice compared to control naïve mice, we performed a Western blot analysis of Ca_v_3.3 protein levels from whole TG lysates (n = 3 mice per group; Figure 2a). We found that signal intensity for Ca_v_3.3 was almost absent in naïve mice and was strongly present in FRICT-ION mice treated with the TAT-C3D control peptide at the time of peak effect (4 hr). We also noticed that Ca_v_3.3 signal intensity in TG of TAT-C3P-injected mice appeared lower than in control TAT-C3D-injected mice (Figure 2a). Finally, to again compare males and females, we calculated the fold change of normalized intensity (compared to actin) of Ca_v_3.3 signal intensity for TAT-C3P-injected mice to TAT-C3D-injected mice between male and female FRICT-ION mice (Figure 2b). We found a significant difference (p < 0.05, t-test) between the fold-changes with female mice versus a smaller fold-change in male mice indicating that TAT-C3P is more effective in reducing Ca_v_3.3 expression in female than in male mice.

## Discussion

*Cacna1i*, encoding the Ca_v_3.3 channel, was 1 of 6 genes encoding an ion channel upregulated 10 weeks post-trigeminal nerve injury in whole TG compared to controls, while many ion channel mRNAs were downregulated at this chronic time point in our model. We also confirmed that Ca_v_3.3 protein levels are elevated in the TG of FRICT-ION mice. We also show that the selective block of Ca_v_3.3 is an effective strategy for alleviating neuropathic pain in both male and female mice in our model. While it may be unusual for RNA levels to not be upregulated as starkly as protein levels for Cav3.3 in the TG of FRICT-ION compared to naïve mice, it is becoming more and more well recognized that RNA expression is not the same as protein expression in biological systems and in some cases can even be inversely correlated [10].

Ca_v_3.3 belongs to the family of T-type Ca^2+^ channels, which are expressed in small- and medium-diameter primary afferent neurons [11], regulate neuronal excitability, and have a well-characterized role in neuropathic pain [12,13]. Several groups have successfully demonstrated that blocking or attenuating all T-type Ca^2+^ channels or, in particular, the Ca_v_3.2 subtype are effective strategies for mitigating behavioral signs of neuropathic pain in animal models [14–18]. In addition, the pan-T-type Ca^2+^ channel blocker ethosuximide has been tested in clinical trials for neuropathic pain [19]. Interestingly, this particular trial was halted due to adverse events experienced by patients, suggesting that more specific Ca_v_3 blockers may be required for neuropathic pain.

With regard to Ca_v_3.3 research, we find only a single study where the team interfered with Ca_v_3.3 channel function using specific antisense oligonucleotides (ASOs) and demonstrated a corresponding mitigation of neuropathic pain behaviors in the chronic compression of the dorsal root ganglion (CCD) model [20]. We also find only a single study implicating T-type Ca^2+^ channels in trigeminal neuropathic pain. In this study, the authors use an infraorbital nerve ligation model of trigeminal neuropathic pain and reveal that Cav3.1 channels are critically involved [21]. Therefore, the present study demonstrates a truly novel role for Ca_v_3.3 channels in a model of trigeminal neuropathic pain.

We unexpectedly found that blocking Ca_v_3.3 with TAT-C3P appears to be more effective in alleviating mechanical allodynia in female mice than male mice. There was no effect of the control TAT-C3D peptide on mechanical allodynia in our model.

Our results further indicate that the TAT-C3P peptide, which selectively inhibits Ca_v_3.3 channels [9], can reduce protein levels of Ca_v_3.3 in the TG of injected mice compared to controls. This suggests that TAT-C3P may help normalize Ca_v_3.3 to baseline levels. We are uncertain as to how the acute blockade of Cav3.3 may translate to downregulation of the protein; however, it is possible that TAT-C3P may be influencing channel trafficking in addition to blocking channel activity as has been observed with other calcium channel-modulating neuropathic pain drugs such as pregabalin [22,23]. TAT-C3P has no effect on Cav3.3 currents when applied acutely to Cav3.3-expressing HEK cells or when dialyzed acutely into cells via the patch pipette, which usually rules out a direct action on the channel [9]. This suggests either an effect on the trafficking/stability of the channel, or potentially an effect on gene expression, which might be consistent with our observation that Cav3.3 levels are decreased upon injection of the TAT-C3P peptide *in vivo*.

The low relative expression of Cav3.3 protein in sham compared to injured mice has been noted previously by others in the spinal cord [20]. The increase at week 10 compared to naïve speaks to the importance of inducible changes in a chronic neuropathic pain model, which might not appear in an acute pain model.

In addition, female mice injected with TAT-C3P displayed a greater decrease of Ca_v_3.3 signal intensity compared to control TAT-C3D peptide than male mice. This infers that the TAT-C3P peptide is more effective in reducing Ca_v_3.3 levels in female mice, which may explain the behavioral sex differences of the TAT-C3P peptide. This is the first example of a sex difference reported for a T-type channel blocking drug for any type of neuropathic pain.

In summary, this study presents a promising case for the development of a novel therapeutic targeting Ca_v_3.3. However, further testing in other models of neuropathic pain and eventually in primates will be required to determine if Ca_v_3.3-blockade is an effective strategy for treatment in patients with trigeminal neuropathic pain.
